# Polarization-independent, wide-incident-angle and dual-band perfect absorption, based on near-field coupling in a symmetric metamaterial

**DOI:** 10.1038/s41598-017-11824-7

**Published:** 2017-09-14

**Authors:** Bui Son Tung, Bui Xuan Khuyen, Young Ju Kim, Vu Dinh Lam, Ki Won Kim, YoungPak Lee

**Affiliations:** 1 Hanyang University, Department of Physics and RINS, Seoul, 04763 Korea; 2 Institute of Materials Science, Vietnam Academy of Science and Technology, Hanoi, Vietnam; 3 Sunmoon University, Asan, 31460 Korea

## Abstract

We numerically and experimentally investigated a dual-band metamaterial perfect absorber (MPA), utilizing the near-field coupling of double split-ring resonators (DSRRs). Owing to the near-field coupling between resonators, two arms in each DSRR resonate in different phases, leading to a dual-band perfect absorption. The proposed MPA also exhibits polarization-insensitive behavior and maintains the high absorption above 90% up to a wide range of incident angle more than 45°. Finally, to further consolidate our approach, a multi-band absorption is also studied by exploiting the near-field coupling among a larger number of DSRRs. Our work is expected to be applied to future broadband devices using MPA.

## Introduction

Metamaterial perfect absorbers (MPAs) have drawn much attention in science society due to potential applications in energy harvester^1^, bio-sensor^2^, subwavelength resolution^3^, photovoltaics^4^, emitter^5^, detector^6^ and modulator^7^. Since the first demonstration of MPA in 2008^8^, many MPAs have been demonstrated in different regions such as MHz^9, 10^, GHz^11, 12^, THz^13, 14^, infrared^15, 16^ and optical^17, 18^ frequencies. For real applications and devices, a broad bandwidth is highly preferred, making multi-band and broadband MPAs one of the main research directions. Although several approaches have been developed for multi-band and broadband absorption so far, specific obstacles still exist. Resistor-integrated MPAs^19^ exploit the loss of resistor to broaden the resonance bandwidth, but the operational frequencies are limited to low frequencies (below GHz) due to the size of resistors. Multi-layer stacking^20^, where different layers yield their own resonance frequencies, is also a way to create multi-band and broadband MPAs. However, the thickness is much increased making the MPA hard to be applied in ultrathin devices. Another similar way is combining many resonators^21^, which share a same design but have small deviations in the geometrical size, to exhibit closely separate resonances in the plane of structure. Unfortunately, in order to be broadband, the deviations of geometrical size should be small enough, which might be impossible for fabrication, especially, in the optical region.

Recently, the near-field coupling in metamaterial (MM), which is famous for electromagnetically-induced transparency (EIT)^22, 23^, has been exploited as a different way to obtain multi-band absorption because the MPA structures do not suffer from the problem of size deviations in the combined-resonator method^24–26^. However, to induce an EIT-like effect for multi-band absorption, an asymmetry of structure should be devised, making the MPA strongly polarization-sensitive. In this report, we propose another approach to create multi-band absorption, based on the near-field coupling in a symmetric MM structure. By exploiting the near-field coupling of identical resonators, the dual-band and the multi-band absorption can be induced. Differently from the mentioned works, the coupling occurs in a symmetric structural design instead of an asymmetric one, for the MPA to be polarization-insensitive. The proposed absorber is not only polarization-insensitive but works for a wide range of incident angle, which is useful for real applications.

## Results

### Design of structure

**Figure 1:**
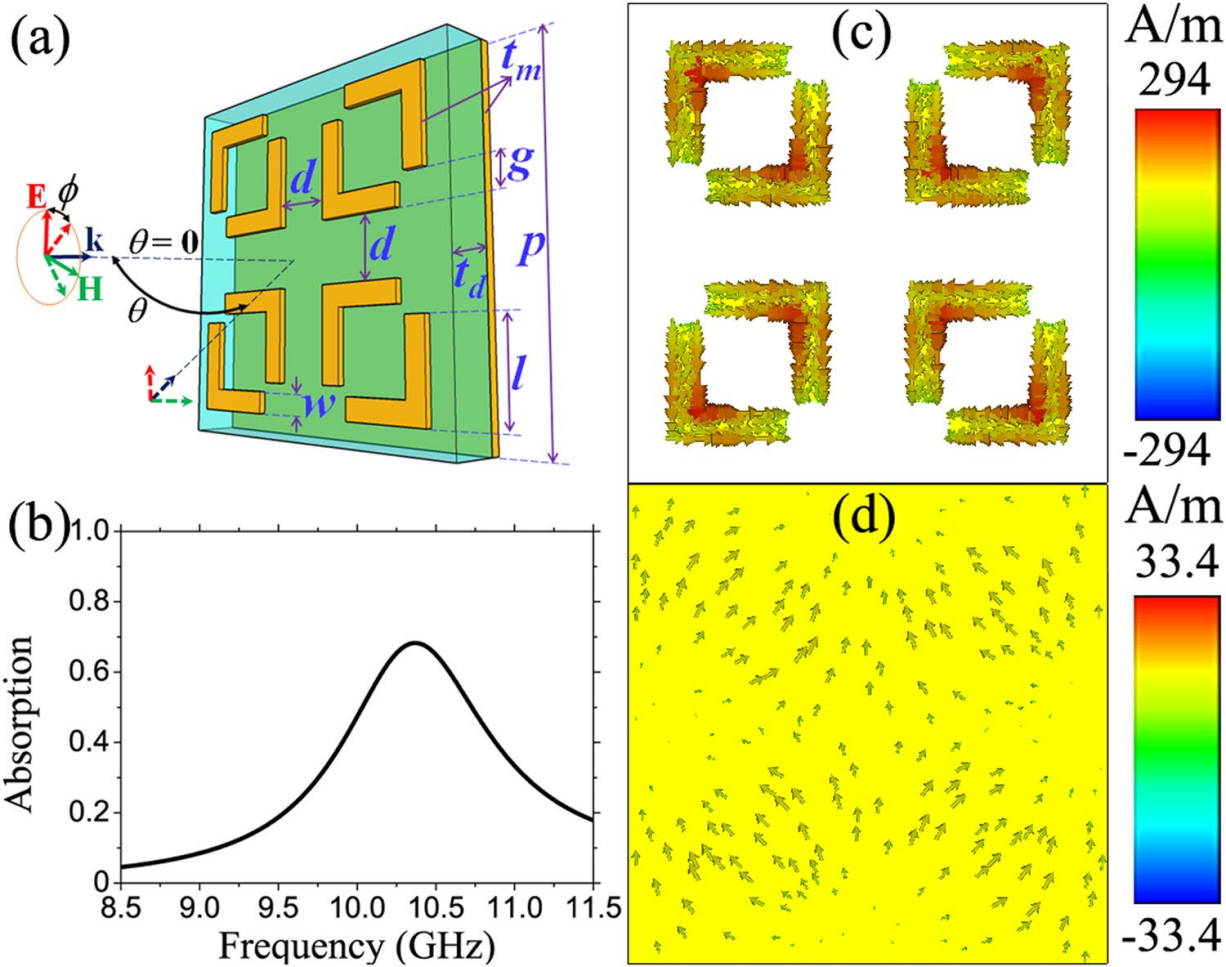
(**a**) Illustration of the unit cell of MPA with the polarization of electromagnetic wave. (**b**) Absorption spectrum of the MPA. Surface currents on (**c**) the front patterned and (**d**) the continuous back metallic layers.

Figure 1(a) presents an illustrated unit cell of our proposed MPA structure, which is composed of a front patterned metallic layer, a middle dielectric spacer and a back metallic film. Copper is chosen as the metal with a conductivity of 5.96 × 10^7^ S/m, while the dielectric layer is made of FR-4 with a dielectric constant of 4.3 and a loss tangent of 0.025. The thicknesses of metallic layers and dielectric spacer are *t*_*m*_ = 0.035 and *t*_*d*_ = 1.6 mm, respectively. The patterned metallic structure consists of four identical double split-ring resonators (DSRRs), which are rotated by 90° in turn to make a symmetric MPA structure and separated from each other by a distance *d* = 3.5 mm. The arms of DSRR have a side length *l* = 4.5 mm and a width *w* = 1 mm and the gap between these arms is *g* = 1.5 mm. The whole MPA is a periodic array of the unit cell with a periodicity *p* = 19 mm. The incident electromagnetic (EM) wave is normal to the MPA plane with electric (**E**) and magnetic (**H**) fields are polarized along the arm lengths and are perpendicular to each other. Figure 1(b) is the simulated absorption spectrum of proposed MPA structure. Because the left and the right DSRRs are mirror-symmetric with respect to the **E** direction, the responses of four rings are identical. Therefore, only one absorption peak is observed at 10.3 GHz with an absorption of 68.2%. The surface currents in Figs 1(c) and (d) reveal that the absorption results from a magnetic resonance, indicated by the anti-parallel currents on the front and the back metallic layers.

### Dual-band metamaterial absorber, based on near-field coupling: fabrication and characterization

**Figure 2:**
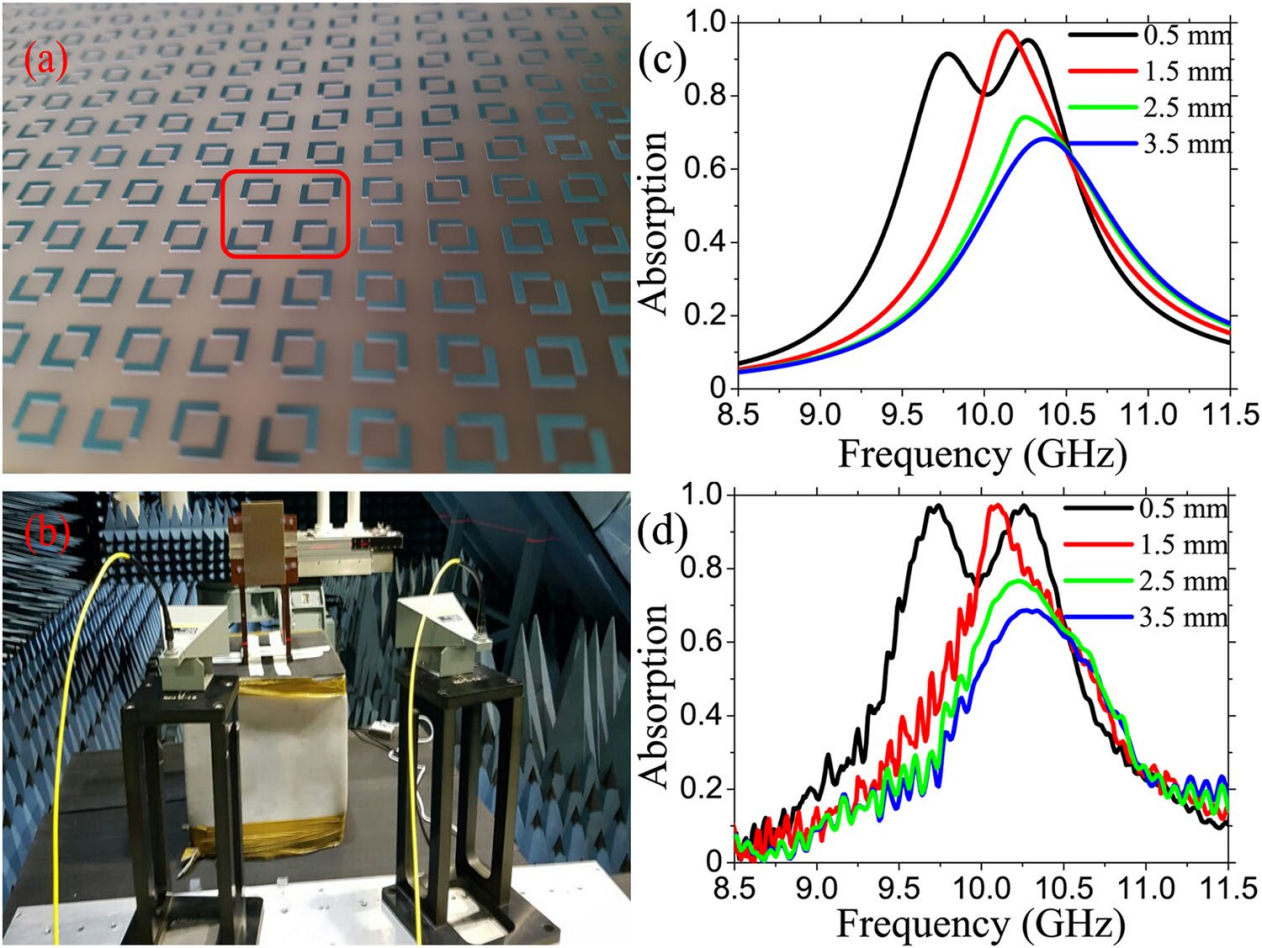
(**a**) A fabricated MPA sample, the red box indicates the unit cell of structure. (**b**) Measurement configuration. (**c**) Simulated and (**d**) experimental absorption spectra according to separation *d* of DSRRs.

Next, an approach to make a dual-band MPA, based on the near-field coupling, is demonstrated. Figures 2(a) and (b) show a fabricated sample and the measurement configuration, respectively. Four samples with the distance *d* varied from 3.5 to 0.5 mm were fabricated by using the traditional printed-circuit board, followed by the photolithography process. Because there is no transmission through MPA due to the back metal film, only the reflection measurement is set up. The absorption is calculated as *A*(*ω*) = 1 − |*S*_11_(*ω*)|^2^, where *S*_11_(*ω*) is the reflection parameters. The simulated and the measured absorption spectra according to *d* are given in Figs 2(c) and (d), respectively, which show a good agreement between them. Initially, when the DSRRs are far from each other, there is only one absorption peak as demonstrated above. When the separation between rings are smaller, the near-field interaction between them is considerable and stronger. Therefore, the shape of absorption spectrum is gradually distorted, becomes asymmetric and, finally, the initial peak splits clearly into two absorption peaks for *d* = 0.5 mm. The absorption peaks are located at 9.7 and 10.3 GHz, both with an absorption of 97.1%.

**Figure 3:**
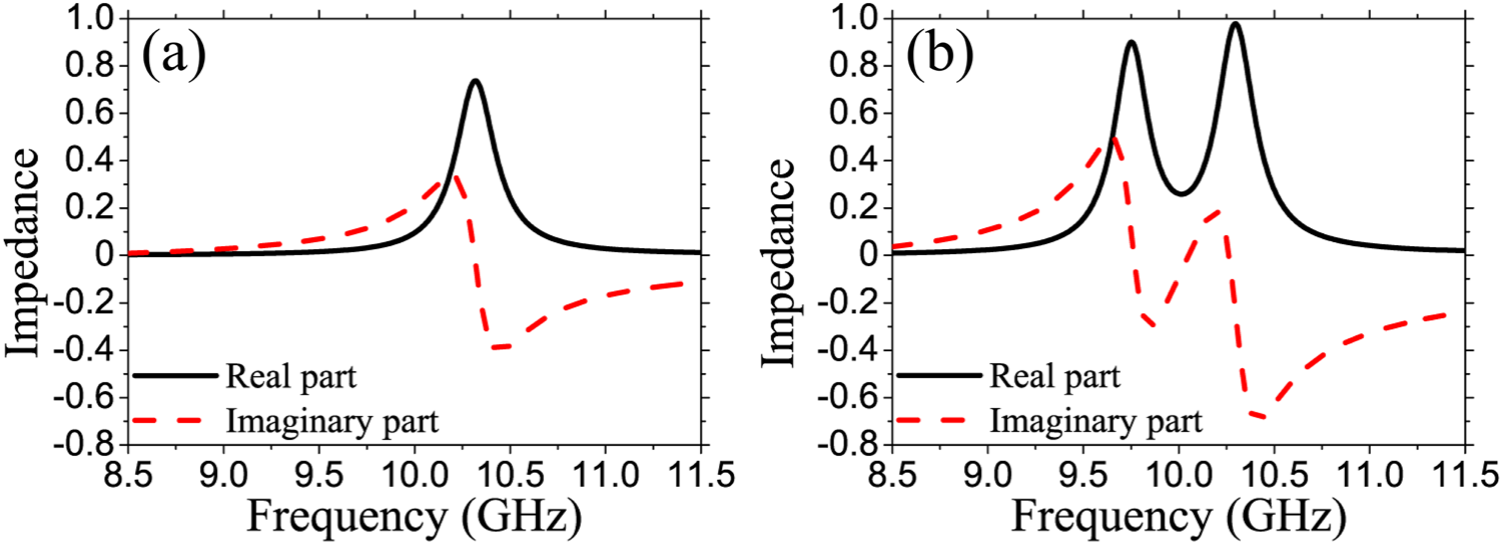
Real and imaginary parts of impedance for (**a**) *d* = 3.5 and (**b**) *d* = 0.5 mm.

The impedance spectra of MPAs for *d* = 3.5 and *d* = 0.5 mm are presented in Fig. 3. The impedance of MPA is calculated using the expression below^27^:

1 $$Z(\omega )=\sqrt{\frac{{\mathrm{(1}+{S}_{11}(\omega ))}^{2}-{S}_{21}{(\omega )}^{2}}{{\mathrm{(1}-{S}_{11}(\omega ))}^{2}-{S}_{21}{(\omega )}^{2}}}$$

The results clarify that the impedance of MPA at two frequencies 9.7 and 10.3 GHz matches well with that of the air for *d* = 0.5 mm. Therefore, the reflection is minimized at these frequencies. Combining with the transmission block by the back metal, consequently, two near-perfect absorption peaks are observed as in Fig. 2. Furthermore, as shown in Fig. 3(a), the impedance spectrum of MPA only have one peak and it is not really well matched, which explains why the absorption is low for *d* = 3.5 mm.

**Figure 4:**
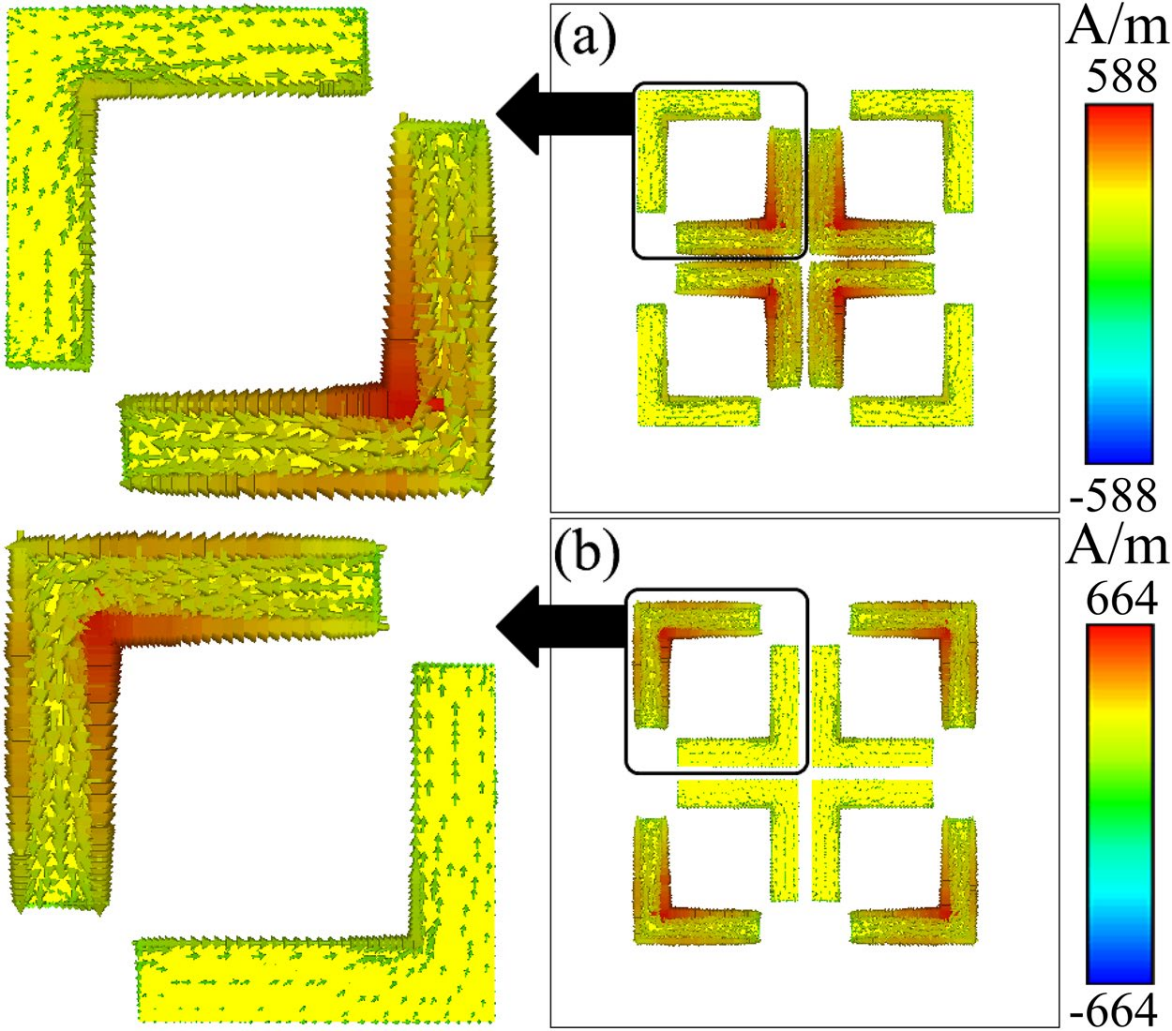
Surface currents on the front patterned metallic layer at (**a**) 9.7 and (**b**) 10.3 GHz when *d* = 0.5 mm. The black boxes and arrows indicate the magnified areas.

To understand better the nature behind the observed phenomena, the surface currents on the front patterned metallic layer are presented at two absorption frequencies, as shown in Fig. 4. Differently from Fig. 1(c) when *d* = 3.5 mm, two arms of DSRRs do not exhibit the same behavior. The induced currents on inner arms are maximized while those on outer arms are nearly minimized at the first resonance [Fig. 4(a)]. On the other hand, at the second resonance, the maximum and the minimum currents are observed on the outer and the inner arms, respectively. In addition, the induced currents on DSRRs are anti-parallel at the two resonance frequencies. Furthermore, at both resonance frequencies, the directions of currents on two arms of individual DSRR are also opposite. These results indicate that the near-field coupling between DSRRs induces highly contrastive states not only between two resonance frequencies but between two arms of individual DSRR at a same resonance frequency. In the attached Supplementary Information, the near-field coupling between DSRRs is described further by the radiating two-oscillator model^28^ for a simplified structure, in which the back metal layer of MPA is removed.

**Figure 5:**
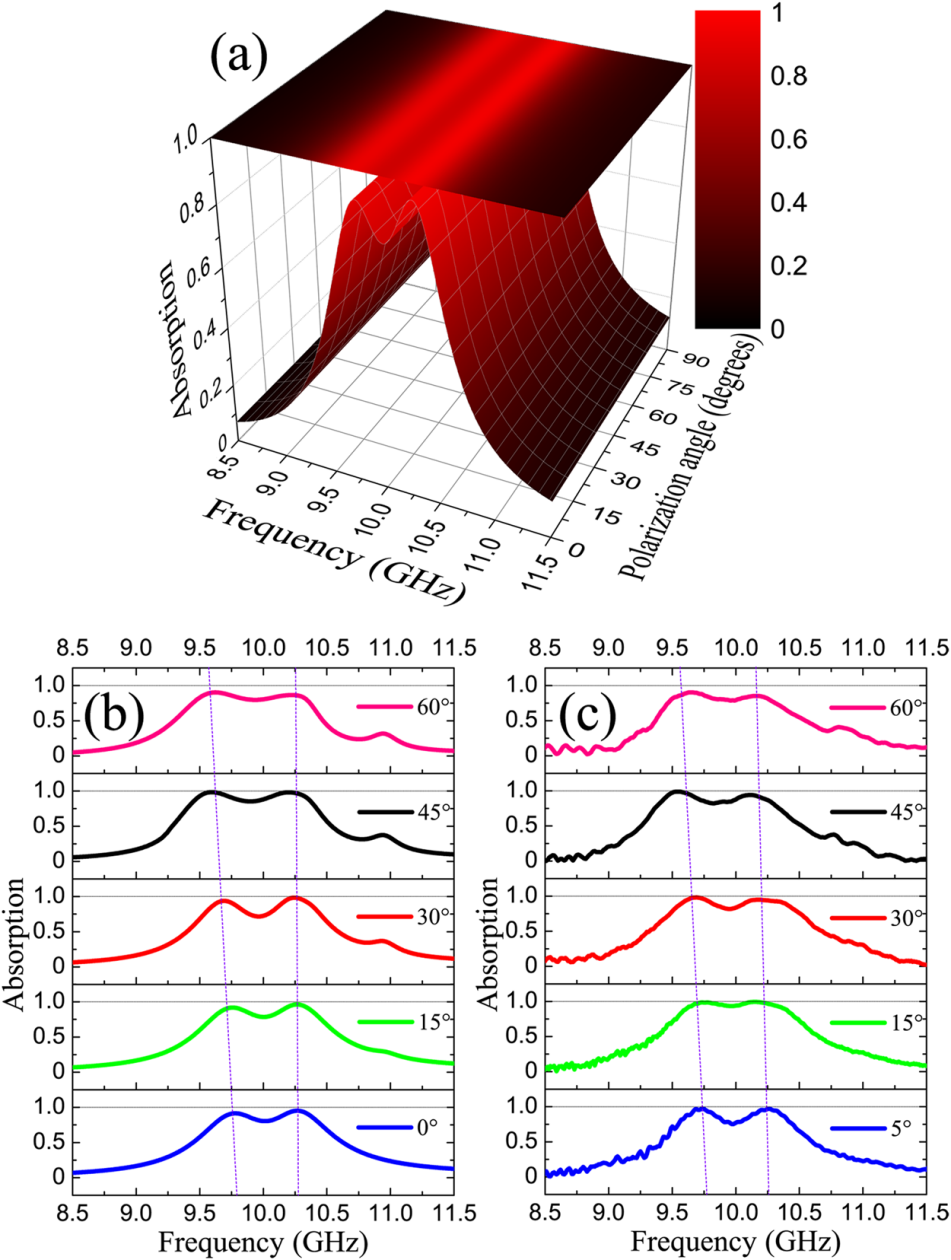
(**a**) Simulated absorption spectra according to polarization angle. Dependence of (**b**) simulated and (**c**) experimental absorption spectra on the incident angle *θ* of EM wave.

To evaluate further the real applicability of MPA, the dependences of polarization and incident angle of EM wave are investigated. As shown in Fig. 5(a), the simulated absorption spectrum of MPA is independent of the polarization state at the normal incidence. The polarization insensitivity of MPA is achieved due to the symmetric geometry of structural design. Figures 5(b) and (c) present the simulated and experimental absorption spectra according to incident angle. Both results are in quite good agreement. The dual-band absorption is maintained above 90% in a wide range of incident angle (*θ* > 45°). At *θ* = 60°, the absorption starts reducing to be 90% and 85% for the first and the second peaks, respectively. It is also shown that the frequency interval between two absorption peaks is slightly larger by increasing the incident angle. The observed phenomenon might be explained, based on the near-field coupling between resonators. When the incidence is not normal to the structural plane, the MPA structure can be considered to be asymmetric with respect to the EM wave^29, 30^. The asymmetric factor also contributes to the near-field coupling between resonators, making the coupling strength stronger. Consequently, the two split resonances are separated further at larger incident angle.

**Figure 6:**
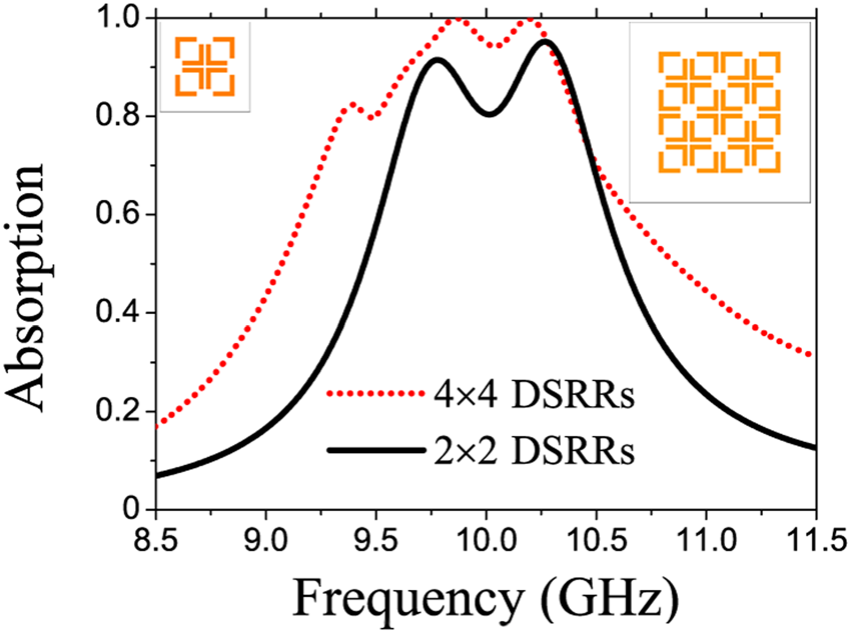
Comparison of the simulated absorption spectra between two MPAs with the unit cells of 2 × 2 (left inset) and 4 × 4 (right inset) DSRRs. The periodicity of 4 × 4 DSRR structure is 38 mm, which is as twice as that of the 2 × 2 DSRR structure.

Finally, to consolidate our proposed approach, an extended structure is presented and compared with the dual-band MPA structure. The extended structure exploits the near-field coupling of a larger number of resonators (4 × 4 DSRRs) than that of the dual-band MPA structure (2 × 2 DSRRs). As shown in Fig. 6, the simulated absorption spectrum of 4 × 4 DSRRs structure is broader and more efficient than that of 2 × 2 DSRRs structure. Instead of the dual-band absorption, the improved MPA exhibits a multi-band absorption (four obviously-observable peaks at least) with the bandwidth at 80% absorption is broader by 37%. Especially, a broadband absorption region, whose absorption is over 90%, is obtained with a bandwidth of 0.5 GHz.

## Discussion

In summary, we demonstrated an approach to make a dual-band MPA by making use of the near-field coupling in a symmetric structure. Both simulated and experimental results show transformation of the absorption spectrum from single- to dual-band absorption by adjusting the separation of DSRRs. The mechanism is clarified to be the near-field coupling, which makes the MPA behave differently at two resonance frequencies, and the inner and the outer arms of DSRRs exhibit opposite behaviors even at the same resonance frequency. The proposed dual-band MPA is also polarization-insensitive and able to operate in a wide range of incident angle. Finally, by extending the number of coupled resonators, we presented that the absorption could be improved to be broader and more efficient. Our work contributes another way to provide broadband MPAs, which might be useful in various future applications.

## Methods

### Simulation

In our work, the simulation was performed by using a finite-integration package (CST Microwave Studio^31^). The unit-cell boundary was applied along two orthogonal directions on the plane of structure. The polarization of electromagnetic wave was chosen as in Fig. 1(a).

### Fabrication

The fabrication was done using the conventional printed-circuit-board method. A FR-4 substrate of 1.6 mm thickness was sandwiched between 0.035 mm-thick copper layers, one of which is coated by photoresist. After putting a mask, which has the design of structure, on one side of sample, the photolithography process was carried out. Then, the exposed part of the copper film was removed by the wet-etching technique, giving the structural pattern.

### Measurement

The measurement was carried out in a microwave anechoic chamber using a Hewlett-Packard E8362B network analyzer connected to linearly-polarized microwave standard-gain horn antennas. In order to fulfill the far-field condition, the distance between antennas and sample was set to be 2.0 m, which is around 66 times larger than the absorption wavelength.

## Electronic supplementary material

Supplementary Information
