# Supplementary material for: Polarization-independent, wide-incident-angle and dual-band perfect absorption, based on near-field coupling in a symmetric metamaterial
Source: Sci Rep. 2017 Sep 14;7:11507. doi: 10.1038/s41598-017-11824-7 (PMC5599624; doi:10.1038/s41598-017-11824-7)
Supplement: Supplementary file 1 — Supplementary Information [file 41598_2017_11824_MOESM1_ESM.pdf]

Supplementary Information for "Polarization-independent, wide-incident-angle and dual-band perfect absorption, based on near-field coupling in a symmetric metamaterial"

Bui Son Tung, Bui Xuan Khuyen, Young Ju Kim, Vu Dinh Lam, Ki Won Kim, and YoungPak Lee

Here, in this Supplementary Information, we present details on the coupling between double split-ring resonators (DSRRs) using the radiating two-oscillator model.

Radiating two-oscillator model for coupled DSRRs

Generally, for a system of two harmonic resonators, coupled with the external field, the electromagnetic behavior of system is governed by the equations below [1, 2].

$$\ddot{x}_1(t) + \gamma_1\dot{x}_1(t) + \omega_1^2 x_1(t) - \tau x_2(t) = E_1(t), \quad (1)$$

$$\ddot{x}_2(t) + \gamma_2\dot{x}_2(t) + \omega_2^2 x_2(t) - \tau x_1(t) = E_2(t), \quad (2)$$

where ω_1 and γ_1 are the resonance frequency and the damping factor, respectively, of the first radiative oscillator, which is described by excitation $x_1(t)$, while ω_2 and γ_2 are the resonance frequency and the damping factor, respectively, of the second radiative oscillator, which is described by excitation $x_2(t)$. These oscillators are under external field $E_1(t)$ and $E_2(t)$, respectively, and τ is the coupling coefficient between oscillators.

By considering that $x_1(t) = D_1 e^{-i\omega t}$, $x_2(t) = D_2 e^{-i\omega t}$ and $E_1(t) = E_2(t) = Ee^{-i\omega t}$, D_1 and D_2 are obtained to be

$$D_1 = \frac{\tau + (\omega_2^2 - \omega^2 - i\omega\gamma_2)}{(\omega_1^2 - \omega^2 - i\omega\gamma_1)(\omega_2^2 - \omega^2 - i\omega\gamma_2) - \tau^2}, \quad (3)$$

$$D_2 = \frac{\tau + (\omega_1^2 - \omega^2 - i\omega\gamma_1)}{(\omega_1^2 - \omega^2 - i\omega\gamma_1)(\omega_2^2 - \omega^2 - i\omega\gamma_2) - \tau^2}. \quad (4)$$

According to the approximation of thin film structures [3], the electric current density J and the surface conductivity σ_e can be expressed as

$$J = -in_s\omega(D_1 + D_2) = \sigma_e E_s, \tag{5}$$

where n_s and E_s are the average electron density and the spatially-averaged electric field, respectively. By assuming that $E \approx E_s$, we have

$$\sigma_e = -in_s\omega \frac{2\tau + (\omega_2^2 - \omega^2 - i\omega\gamma_2) + (\omega_1^2 - \omega^2 - i\omega\gamma_1)}{(\omega_1^2 - \omega^2 - i\omega\gamma_1)(\omega_2^2 - \omega^2 - i\omega\gamma_2) - \tau^2}. \tag{6}$$

Finally, the reflection and the transmission coefficients of metamaterial (MM) have the form of

$$R = \frac{-Z_0\sigma_e}{2 + Z_0\sigma_e}, \tag{7}$$

$$T = \frac{2}{2 + Z_0\sigma_e}, \tag{8}$$

, respectively, where Z_0 is the wave impedance of external wave.

In order to clarify the nature of dual-band absorption, the coupling behavior of 4 DSRRs is considered. The investigated MM has only two layers, the patterned layer (DSRR structure) and the FR-4 substrate. There is no back metallic layer. The geometrical parameters of MM are similar to those of the proposed absorber. In this MM structure, two sets of two DSRRs can be considered to be a bright-mode oscillator and the total interaction behavior can be presented by the radiating two-oscillator model. Figure S1 presents the simulated transmission of investigated MM for two cases of d = 3.5 and 0.5 mm. Initially, when the DSRRs are far from each other, there is only one resonance. By decreasing the distance between rings, the initial resonance splits into two separated resonances. This phenomenon indicates a near-field coupling between DSRRs. The radiating two-oscillator model is applied to describe the interaction for the case of d = 0.5 mm. The calculated transmission around the coupling frequency region is also plotted in Fig. S1 by fitting Eq. 8 to the simulated transmission. The calculated result is in agreement with the simulated result at frequencies around the coupling region. According to our calculation, the coupling coefficient τ is 18, and the damping factors of resonators γ_1 and γ_2 are 0.12 GHz and 0.55 GHz, respectively. At the lower and the higher frequencies, there are differences between simulation and calculation. The mismatch might be due to the dispersive nature of substrate, which is not

accounted in the theoretical model. Besides, the high-order resonance modes might also affect the behavior of system.

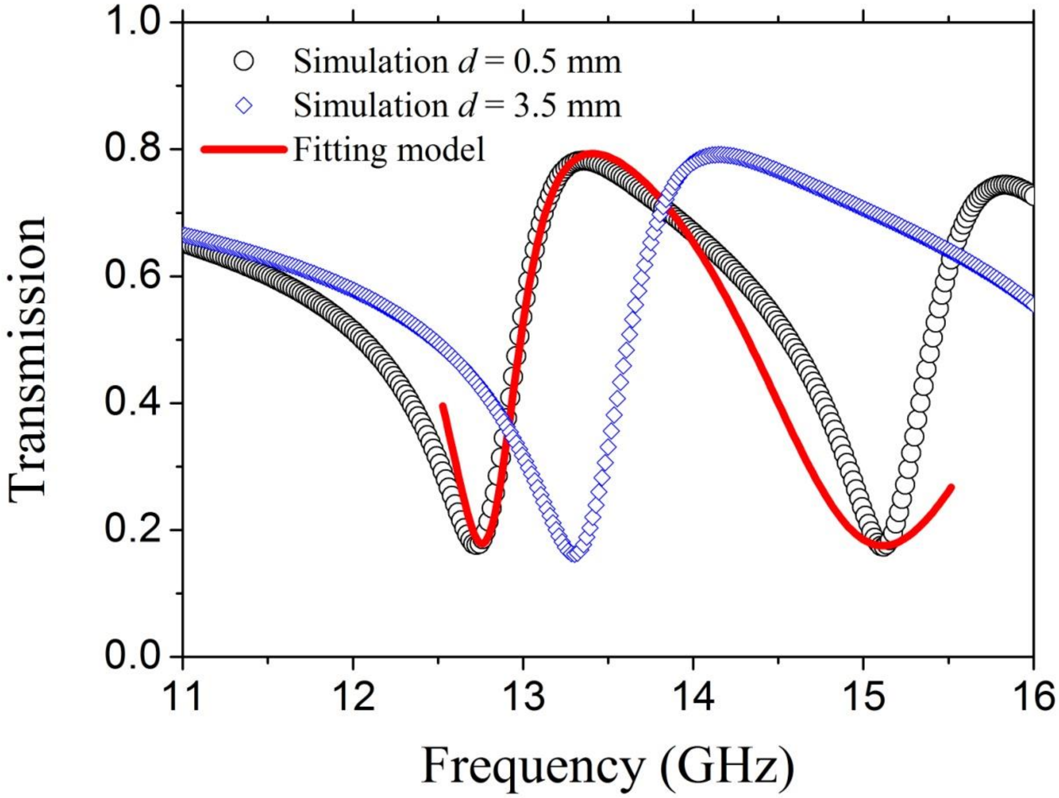

Figure S1. Simulated transmission spectra of DSRR structure for different distance d between DSRRs. The fitted transmission is calculated for the case $d = 0.5$ mm, based on the radiating two-oscillator model.

References

[1] Tassin, P. *et al.* Electromagnetically induced transparency and absorption in metamaterials: the radiating two-oscillator model and its experimental confirmation. *Phys. Rev. Lett.* **109**, 187401 (2012).

[2] Hu, X. *et al.* Plasmon induced transparency and absorption in bright–bright mode coupling metamaterials: a radiating two-oscillator model analysis. *J. Phys. D: Appl. Phys.* **50** 025301 (2017).

[3] Tassin, P., Koschny, T., Soukoulis, C. M. Effective material parameter retrieval for thin sheets: Theory and application to graphene, thin silver films, and single-layer metamaterials. *Physica B* **407** 4062-4065 (2012).
